# Correlation of Disease Activity Scores and Routine Assessment of Patient Index Data (RAPID3) on a Multidimensional Health Assessment Questionnaire in Patients With Rheumatoid Arthritis: A Saudi Experience

**DOI:** 10.7759/cureus.44982

**Published:** 2023-09-10

**Authors:** Sultana Abdulaziz, Ahmed S Almaqati, Kalthom Komo, Abdullah Alahmadi

**Affiliations:** 1 Department of Medicine/Rheumatology, King Fahad General Hospital, Jeddah, SAU; 2 Department of Internal Medicine/Rheumatology, King Abdulaziz Medical City - Jeddah, Jeddah, SAU; 3 Department of Internal Medicine/Rheumatology, King Abdullah International Medical Research Center, Jeddah, SAU

**Keywords:** quality of life, disease activity, rheumatoid arthritis, rapid, prom

## Abstract

Introduction

This study aimed to analyze the correlation between Routine Assessment of Patient Index Data (RAPID3) and disease activity scores using the Disease Activity Score 28 (DAS28 erythrocyte sedimentation rate (ESR)/C-reactive protein (CRP)), Clinical Disease Activity Index (CDAI), and Simplified Disease Activity Index (SDAI) in patients with rheumatoid arthritis (RA) attending a single rheumatology center in Jeddah.

Methods

A cross-sectional study of patients with RA who fulfilled the 2010 American College of Rheumatology criteria for the diagnosis of RA between June 2018 and November 2019 was conducted. The validated Arabic version of the multidimensional health assessment questionnaire (MDHAQ) was used. The data collected included demographic information, comorbid illnesses, concomitant medications, laboratory results, and disease activity measured using the DAS 28 ESR/CRP, CDAI, SDAI, and RAPID3.

Results

A total of 137 patients with RA were included in the analysis; the mean age was 53.1 (± 12) years, there were 122 (89%) females, and the mean disease duration was 8 (± 4) years. Forty-nine (44.5%) patients were treated with anti-tumor necrosis factor (anti-TNF), 53 (48.2%) with non-anti-TNF, 8 (7.3%) with Janus kinase (JAK) inhibitors, and 27 (20%) with synthetic disease-modifying drugs (sDMARD).

The mean RAPID3 (0-10) score was 3.6 (± 2) for low disease activity. The mean DAS28 ESR was 4.16 (± 4) for moderate disease activity. The mean DAS CRP was 3.39 (± 1.2) for moderate disease activity. The mean CDAI was 13.4 (± 10.7) for moderate disease activity. The mean SDAI was 15.34 (± 11.8) for moderate disease activity. Pearson’s correlations showed a strong correlation with DAS28 CRP (r=0.773, p < 0.001), SDAI (r=0.764,p < 0.001), CDAI (r=0.710, p < 0.001), and DAS28 ESR (r=0.283, p < 0.002).

Conclusion

RAPID3 significantly correlated with DAS28-CRP, SDAI, and CDAI scores in our patients. It is a simple, inexpensive, and patient-centered practical tool for assessing disease activity that can reflect the health-related quality of life and be easily implemented in clinical practice.

## Introduction

Rheumatoid arthritis (RA) is associated with joint damage, loss of joint function, and reduced quality of life. Therefore, early and aggressive initiation of treatment has been enforced as a paramount step in management [1]. Treat-to-target strategies that focus on targeting remission or low disease activity have been enforced as an overarching principle in multiple guidelines addressing the management of autoimmune rheumatic diseases, especially RA because doing so has reportedly improved outcomes and avoided complications [2-5]. Achieving these treatment goals requires frequent disease activity assessments using different well-validated composite measures that have been recommended as the gold standard for objective evaluation [6].

These clinical disease activity scores were developed by integrating different clinical and/or laboratory results to classify patients as being in remission, with low, moderate, or high disease activity, which, in turn, mandates adjusting the treatment plan according to the score results aimed at achieving remission or low disease activity [7]. Over the last two decades, these scores have been validated, frequently recognized, and implicated in daily clinical practice, including the Disease Activity Score with 28-Joint Counts (DAS28), Clinical Disease Activity Index (CDAI), and Simplified Disease Activity Index (SDAI) [8-10]. However, these measures involve joint count performance in daily clinical practice, which is time-consuming, even though it provides an objective assessment; unfortunately, it was minimized recently during the unprecedented COVID-19 pandemic.

Therefore, with the recent advocacy for physician-patient shared decisions, the need for more practical and patient-centered tools for monitoring disease activity is essential. Patient-Reported Outcome Measures (PROMs) have been advanced alongside clinical disease activity scores in clinical practice to monitor disease activity. PROMs focus on addressing patients’ views of their disease status, response to therapy, and effects of the disease on their quality of life. These scores include but are not limited to the Health Assessment Questionnaire (HAQ), Modified HAQ (MHAQ), Rheumatoid Arthritis Disease Activity Index (RADAI), and Routine Assessment of Patient Index Data 3 (RAPID3), which all have been used in clinical practice [11-13].

Consequently, this study aimed to analyze the correlation between RAPID3 and disease activity scores (DAS28, CDAI, and MHAQ) in patients with RA at a single rheumatology center in Jeddah, Saudi Arabia.

## Materials and methods

This was a cross-sectional retrospective cohort study. All adult patients aged 14 years and older who met the American College of Rheumatology 2010 criteria for the diagnosis of RA who attended the rheumatology outpatient clinic at the King Fahad General Hospital, Jeddah, Saudi Arabia, between July 2018 and 2019 were included in the study. Demographic information, including age, sex, body mass index (BMI), and clinical variables, including disease duration, laboratory tests, medications, and comorbidities were collected from medical records. Moreover, we collected data on the treatment received, which included conventional and synthetic disease-modifying antirheumatic drugs (DMARDs), glucocorticoids, biologics, and Janus kinase (JAK) inhibitors. Disease activity was assessed using four methods: RAPID3, DAS28, CDAI, and SDAI. As part of routine outpatient clinic management, patients underwent a PROMs assessment using the MHAQ by themselves or with the help of a patient educator, if needed, and a validated Arabic version of the questionnaire was used [14]. These methods were analyzed quantitatively as continuous variables (scores) and clinically as categorical variables (remission, mild, moderate, and high). Missing data, which reflected whether the patients had recent laboratory results, were not included in our database. An expert rheumatologist carefully estimated the formal joint counts.

Statistical analysis

IBM SPSS version 20 (IBM Corp, Armonk, NY, USA) was used for data analysis, and a p-value of <0.05 was considered significant. Descriptive results were expressed as mean ± standard deviation (SD), and categorical results were expressed as frequencies and percentages. Differences between categorical variables were tested using Pearson’s chi-squared test to ascertain the strength and direction of the relationship between the scores of each method used to assess RA activity.

Descriptive analyses (frequency, percentage, and SD) were performed for demographic and clinical variables. A Pearson product-moment correlation was used to ascertain the strength and direction of the relationship between the scores of each method used to assess RA activity. Accordingly, the scores were used as continuous variables. To provide better clinical significance, a chi-squared test for association was conducted between the same methods used to assess disease activity; however, for these analyses, the variables were treated as categorical. Whenever the assumptions of the chi-square test were violated, Fisher's exact test was used.

Logistic regressions were adjusted for age, sex, years of RA, and disease activity assessed using DAS28.

Ethical considerations

The study was conducted according to the Declaration of Helsinki, and it was granted IRB approval by the Research and Studies Department - Jeddah Health Affairs (registration number H-02-J-002). All patients provided written informed consent before recruitment. The collected data were stored on a secure institutional computer device to maintain confidentiality.

## Results

Out of 137 patients with RA included, 122 (89.1%) were female, with a mean age ± SD of 53.1 ± 12 years and a mean ± SD disease duration of 8 ± 4 years. Forty-nine (39%) patients were treated with anti-tumor necrosis factor (TNF), 58 (39%) with non-TNF, eight (6%) with JAK inhibitors, and 27 (20%) only with DMARDs. The mean BMI was 30.47 (10.1) kg/m^2^. Self-reported sleep disturbance was reported in 44 (32.1%) patients, anxiety in 56 (40.9%), and depression in 38 (27.7%). Baseline characteristics are presented in Table 1.

**Table 1:** Demographic characteristics of patients with rheumatoid arthritis Descriptive data are presented as frequency, percentage, and SD. SD, standard deviation; BMI, body mass index; TNF, tumor necrosis factor; JAK, Janus kinase; DMARDs, disease-modifying drugs; ESR, erythrocyte sedimentation rate; and CRP, C-reactive protein

	Total (n=137)
Age, mean±SD (years)	53.1±12
Females, n (%)	122 (89.1)
Disease duration, mean±SD (years)	8.07±3.9
BMI, mean±SD (kg)	30.47±10.1
Obesity (>30 kg), n (%)	63 (47.7)
Present treatment	
Anti-TNF n (%)	49 (35.6)
Non-anti-TNF n (%)	53 (38.7)
JAK Inhibitors n (%)	8 (5.8)
DMARDs, n (%)	27 (19.7)
ESR, mean±SD (mm)	30.58±24
CRP, mean ± SD (mg/dL)	9.47±9.2

The mean RAPID3 score ± SD, which ranges from 0 to 10, was 3.6±2 for low disease activity. The mean DAS28-ESR score ± SD was 4.16±4 for moderate disease activity. The mean DAS28 CRP ± SD was 3.39±1.2 for moderate disease activity. The mean CDAI score ± SD was 13.4±10.7 for moderate disease activity. The mean SDAI ± SD was 15.34 ± 11.8 for moderate activity. Detailed results are depicted in Table 2.

**Table 2:** Disease activity of patients with rheumatoid arthritis (DAS28 ESR/CRP, CDAI, and SDAI) Descriptive data are presented as frequency, percentage, and SD. DAS28 ESR/CRP, CDAI, and SDAI indicated moderate disease activity. RAPID3, Routine Assessment of Patient Index Data; DAS28, Disease Activity Score 28; ESR, erythrocyte sedimentation rate; CRP, C-reactive protein; CDAI, Clinical Disease Activity Index; and SDAI, Simplified Disease Activity Index

	Total n:137
RAPID3, mean±SD	3.6±2
DAS28 ESR, mean±SD	4.16±4
DAS28 CRP, mean±SD	3.39±1.2
CDAI, mean±SD	13.42±10.7
SDAI, mean±SD	15.34±11.8
Sleep difficulty, n (%)	44 (32.1)
Anxiety, n (%)	56 (40.9)
Depression, n (%)	38 (27.7)

RAPID3 had the strongest association with SDAI (V=0.536, p<0.001), followed by CDAI (V=0.532, p<0.001), DAS28 CRP (V=0.463, p<0.001), and DAS28 ESR (V=0.443, p<0.001) (Table 3).

**Table 3:** Relationship of RAPID3 with DAS28 ESR/CRP, CDAI, and SDAI Descriptive data are presented as frequency, percentage, and SD. * p-value <0.05 is considered significant. DAS28, Disease Activity Score 28; ESR, erythrocyte sedimentation rate; CRP, C-reactive protein; CDAI, Clinical Disease Activity Index; and SDAI, Simplified Disease Activity Index

Variables		Total	RAPID3				p-value*
			Remission	Low	Moderate	High	
DAS 28 ESR	Remission	20	6 (30.0%)	9 (45.0%)	5 (25.0%)	0 (0.0%)	<0.001^a^
	Low	21	4 (19.0%)	5 (23.8%)	9 (42.9%)	3 (14.3%)	
	Moderate	64	1 (1.6%)	6 (9.4%)	32 (50.0%)	25 (39.1%)	
	High	17	0 (0.0%)	0 (0.0%)	0 (0.0%)	17 (100.0%)	
DAS 28 CRP	Remission	33	11 (33.3%)	13 (39.4%)	8 (24.2%)	1 (3.0%)	<0.001^a^
	Low	25	1 (4.0%)	7 (28.0%)	10 (40.0%)	7 (28.0%)	
	Moderate	64	0 (0.0%)	1 (1.6%)	31 (48.4%)	32 (50.0%)	
	High	11	0 (0.0%)	0 (0.0%)	0 (0.0%)	11 (100.0%)	
CDAI	Remission	22	10 (45.5%)	5 (22.7%)	4 (18.2%)	3 (13.6%)	<0.001^a^
	Low	32	3 (9.4%)	15 (46.9%)	12 (37.5%)	2 (6.3%)	
	Moderate	61	1 (1.6%)	1 (1.6%)	34 (55.7%)	25 (41.0%)	
	High	22	0 (0.0%)	0 (0.0%)	0 (0.0%)	22 (100.0%)	
SDAI	Remission	18	9 (50.0%)	4 (22.2%)	4 (22.2%)	1 (5.6%)	<0.001^a^
	Low	34	4 (11.8%)	16 (47.1%)	13 (38.2%)	1 (2.9%)	
	Moderate	62	0 (0.0%)	1 (1.6%)	31 (50.0%)	30 (48.4%)	
	High	20	0 (0.0%)	0 (0.0%)	1 (5.0%)	19 (95.0%)	

**Figure 1:**
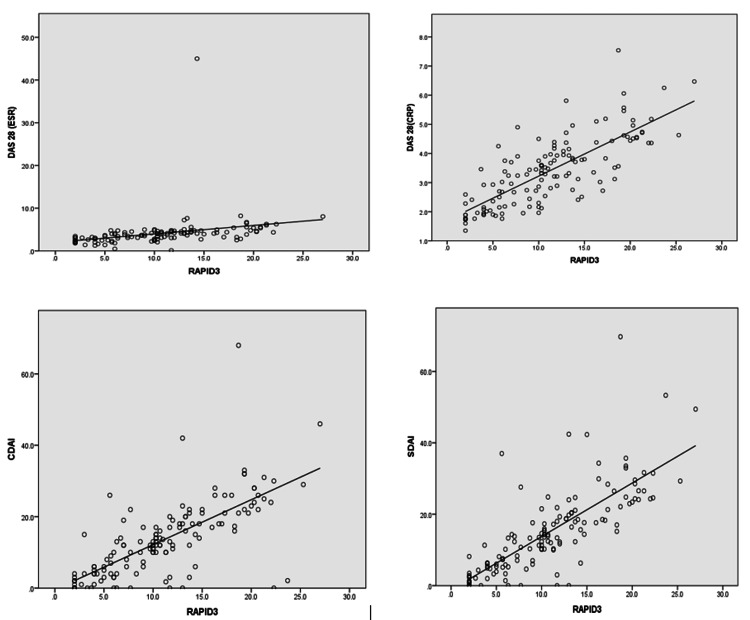
Scatter plot showing the linear regression model between RAPID3 and DAS28-ESR and the CRP, CDAI, and SDAI disease activity scores, which showed strong relationships of DAS28-CRP, CDAI & SDAI with RAPID3 DAS28, Disease Activity Score 28; ESR, erythrocyte sedimentation rate; CRP, C-reactive protein; CDAI, Clinical Disease Activity Index; and SDAI, Simplified Disease Activity Index

The Pearson chi-squared test showed a strong correlation with DAS28 CRP (r=0.77, p<0.001), followed by SDAI (r=0.76, p<0.764), CDAI (r=0.710, p<0.001), and DAS28 ESR (r=0.283, p<0.002) (Figure 1).

Results are presented in detail in Table 4.

**Table 4:** Pearson's correlation coefficient showed the dependency of RAPID3 on disease activity scores Pearson's correlation coefficient r showed a strong correlation with DAS28 CRP (r:0.773, P< 0.001), followed by SDAI (r:0.764,p< 0.001), followed by CDAI (r:0.710,p< 0.001), and then DAS28 ESR (r:0.283,p< 0.002). Correlation coefficient r: -1 negative correlation; 0 no correlation; +1 positive correlation * Correlation is significant at the 0.01 level (2-tailed). ** p-value <0.05 is considered significant. DAS28, Disease Activity Score 28; ESR, erythrocyte sedimentation rate; CRP, C-reactive protein; CDAI, Clinical Disease Activity Index; and SDAI, Simplified Disease Activity Index

Correlations		RAPID3
DAS 28 ESR	r	0.283*
	p-value**	0.002
	N	122
DAS 28 CRP	r	0.773*
	p-value	<0.001
	N	133
CDAI	r	0.710*
	p-value	<0.001
	N	137
SDAI	r	0.764*
	p-value	<0.001
	N	134

## Discussion

The current study assessed disease activity utilizing commonly used scores in daily practice, reflecting real-world data from Saudi Arabia in patients with RA.

Our study demonstrated that RAPID3 strongly correlated with DAS28-CRP, SDAI, CDAI, and DAS28-ESR in patients with RA. This, while not underestimating the importance of a thorough physical examination in daily clinical practice, makes applying RAPID3 in busy outpatient care settings a more practical, time-efficient, and contact-free tool relative to other disease activity scores. Additionally, RAPID3 has the advantage of assessing the patient’s overall complaints, especially those not addressed in the DAS, SDAI, and CDAI scores such as foot pain. Another benefit is the lack of laboratory assessments, including CRP and ESR, which help rheumatologists in rural areas with limited financial resources make more appropriate assessments.

Although our cohort study was conducted in a single medical care center in the Western Region of Saudi Arabia, the results are in agreement with previously reported results. For example, in a study by Muñoz et al., similar correlation rates were found despite having an older mean age (61 vs. 53 years) and a longer disease duration (14 vs. 8 years) [15]. Interestingly, another cohort study conducted in Ecuador with similar demographics, including mean age, disease duration, and sex distribution, concluded that RAPID3 was best correlated with DAS28, CDAI, and SDAI [16].

Moreover, an Indian study by Kumar et al. studied the correlation of DAS28 with other indices, including RAPID3, at the initial presentation of newly diagnosed treatment-naïve patients with RA and found a significant positive correlation between DAS28 and RAPID3; meanwhile, the agreement was less robust [17].

Finally, the results show that RAPID3 is an easily applied patient-centered tool that can be used to assess disease activity in patients with RA, given its correlation with previously described disease activity scores.

Our study had a few limitations. First, it has a small sample size from a single tertiary care center, making the assessment of these measures cautious. Therefore, further large cohort studies are required. Second, elderly patients with difficulty reading require assistance in answering the questionnaire, which necessitates assigning trained healthcare workers for help.

## Conclusions

The study compared the performance of disease activity measurement utilizing different scores. The results showed that the RAPID3 score is significantly correlated with DAS28-CRP, SDAI, CDAI, and DAS28-ESR. RAPID3 is a simple, inexpensive, and patient-centered practical tool for assessing disease activity that reflects the health-related quality of life of patients and can be easily implemented in clinical practice.

The RAPID3 score, which mainly focuses on PROMs, significantly correlated with DAS28-CRP, SDAI, CDAI, and DAS28-ESR. It is a simple, inexpensive, and patient-centered practical tool for assessing disease activity that reflects the health-related quality of life of patients and can be easily implemented in clinical practice. Applying such measures in daily practice can be a time-efficient way that helps rheumatologists assess disease activity faster than other scores without compromising patient care. However, clinicians should consider the strengths and limitations of each scoring system when assessing RA disease activity, basing their decision on the patient’s characteristics and feasibility. Further real-world studies are warranted to validate these findings.
